# A Rare Case of Secondary Angle Closure Glaucoma Due to Soemmering’s Ring in a Pseudophakic Eye: The Role of Ultrasound Biomicroscopy

**DOI:** 10.7759/cureus.103104

**Published:** 2026-02-06

**Authors:** Eleni Papafotiou, Konstantinos Flindris, Chrysa Chatzipetrou, Petros Asteris, Athanasios Kaliardas, Ioannis Koumpoulis

**Affiliations:** 1 Ophthalmology, General Hospital of Ioannina "G. Hatzikosta", Ioannina, GRC; 2 College of Medicine, Aristotle University of Thessaloniki, Thessaloniki, GRC; 3 Ophthalmology, University Hospital of Ioannina, Ioannina, GRC

**Keywords:** angle closure glaucoma, pseudoexfoliation, pseudophakic, soemmering’s ring, ultrasound biomicroscopy

## Abstract

Secondary angle closure glaucoma in pseudophakic eyes is uncommon. Soemmering’s ring, a circumferential proliferation of retained lens material, can, under certain conditions, lead to significant anterior segment distortion and angle closure. Pseudoexfoliation syndrome (PEX) can contribute, through zonular instability and chronic subclinical inflammation, to the anterior displacement of the capsular bag and iris-lens diaphragm. A 70-year-old male with a history of bilateral phacoemulsification presented with progressive visual decline and mild ocular discomfort in the left eye. Slit-lamp examination revealed pseudoexfoliation material on the pupillary margin and a markedly shallow anterior chamber. Intraocular pressure (IOP) was 35 mmHg, and best-corrected visual acuity was 20/100. Despite two patent laser peripheral iridotomies and maximal topical/systemic IOP-lowering therapy, the IOP remained elevated, and the angle remained narrow. Anterior segment optical coherence tomography (AS-OCT) showed anterior bowing of the iris without signs of pupillary block. Ultrasound biomicroscopy (UBM) revealed a 360-degree hyperechoic structure, consistent with an enlarged Soemmering’s ring, in direct contact with the posterior iris surface. The findings indicated a non-pupillary block mechanism of angle closure, likely driven by zonular laxity and capsular displacement. Surgical intervention was eventually required. This case highlights an uncommon but clinically significant mechanism of secondary angle closure in pseudophakic eyes with PEX. Enlargement of a Soemmering’s ring, in combination with zonular instability, can lead to anterior displacement of the iris-lens complex and persistent angle closure, even in the presence of a patent iridotomy. UBM is essential in identifying such atypical mechanisms and guiding effective treatment strategies.

## Introduction

Secondary angle closure in pseudophakic eyes is an infrequent condition that arises from a variety of complex pathophysiologic mechanisms [1-3]. Pupillary block is the most common cause, often occurring when the pupillary aperture becomes occluded by the intraocular lens (IOL), the anterior hyaloid face, or through the formation of posterior synechiae between the iris and the lens optic, which leads to iris bombé [3-5]. Another significant cause is malignant glaucoma (aqueous misdirection), a multifactorial disorder where an abnormal vitreo-ciliary relationship diverts aqueous humor into the vitreous cavity, pushing the lens-iris diaphragm forward and uniformly shallowing the anterior chamber [4-7]. Furthermore, the proliferation of residual lens epithelial cells into a Soemmering’s ring can induce closure through multiple pathways, such as direct mechanical compression of the peripheral iris into the angle, creating a plateau-like iris configuration, or by providing posterior support that maintains apposition even after a patent iridotomy [1-3,8]. Ultrasound biomicroscopy (UBM) is considered essential for accurate diagnosis in pseudophakic eyes, as it allows for the visualization of structures posterior to the iris, enabling clinicians to differentiate between these mechanisms and determine appropriate interventions like laser capsulotomy, iridotomy, or surgical removal of the Soemmering’s ring [1,3,5,9].

Zonular instability in Pseudoexfoliation syndrome (PEX) contributes to angle closure primarily by allowing the pathologic anterior displacement of the lens or the IOL-capsule complex [3,10]. In PEX, abnormal fibrillar material accumulates on the zonules, causing them to become frayed, broken, and progressively weak [10]. This weakened support permits the lens-iris diaphragm to shift forward, which increases iridocorneal contact and facilitates pupillary block [10].

We describe a patient who had progressive angle closure without pupillary block, due to an enlarging Soemmering's ring after phacoemulsification. In addition, we conducted a literature review on the role of Soemmering’s ring in pseudophakic angle closure, with particular emphasis on eyes with PEX.

## Case presentation

**Figure 1:**
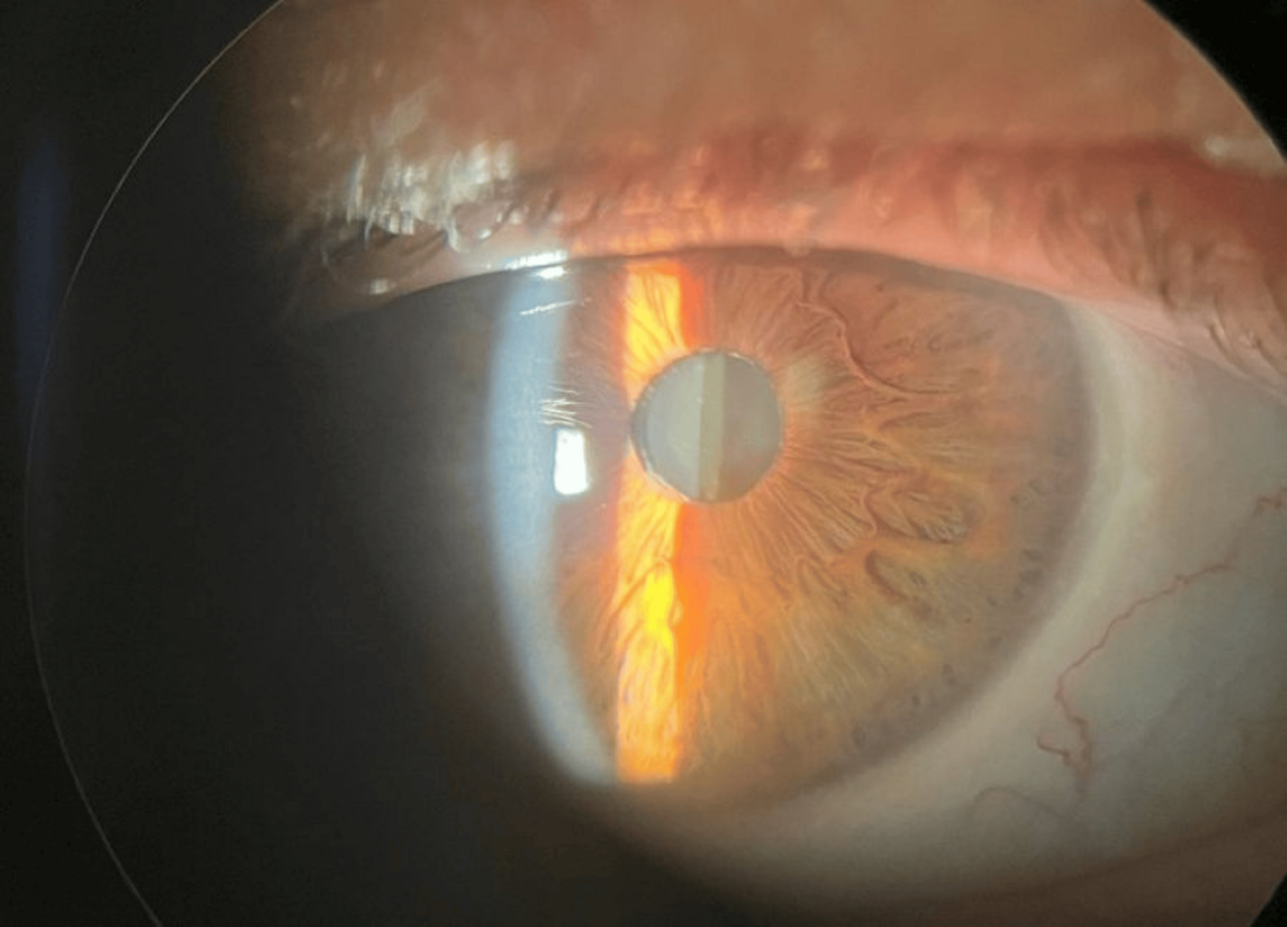
Slit-lamp photograph of the fellow eye showing PEX material deposition at the pupillary margin PEX, pseudoexfoliation

A 70-year-old male presented with visual deterioration in the left eye and ocular pain. He had a history of phacoemulsification with IOL implantation in both eyes five years prior. The patient exhibited pseudoexfoliative material in both eyes, characterized by whitish fibrillary material on the pupillary margin (Figure 1).

**Figure 2:**
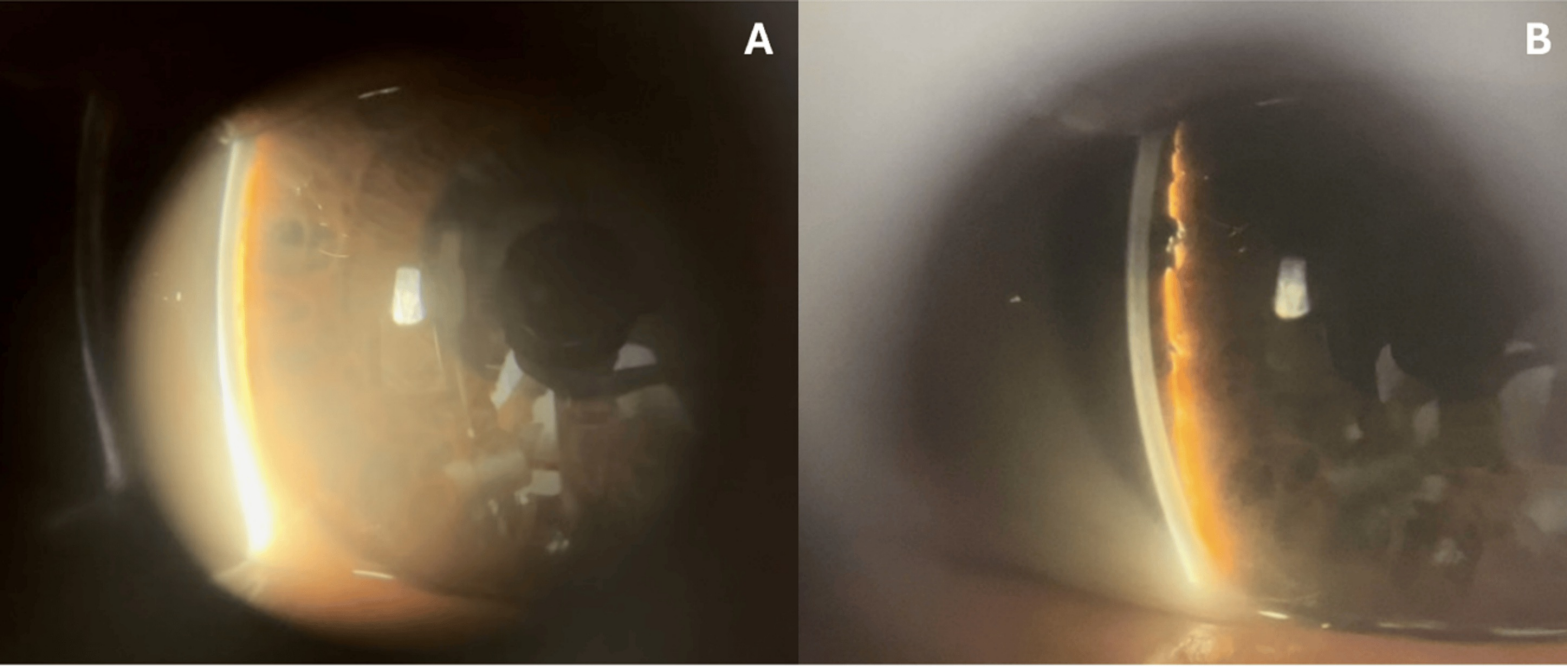
Slit-lamp photographs of the affected eye following LPI at the nine and three o’clock positions (A) A narrow anterior chamber is visible on direct illumination, with the iris appearing to almost touch the cornea peripherally. (B) Shallow anterior chamber angle with a plateau-like configuration despite iridotomy. LPI, laser peripheral iridotomy

Best-corrected visual acuity was 20/20 in the right eye (OD) and 20/100 in the left eye (OS). Intraocular pressure (IOP) was 14 mmHg OD and 35 mmHg OS. Slit-lamp examination of the right eye was unremarkable, while the OS had a shallow anterior chamber, more prominent at the periphery, and corneal edema. Gonioscopy of the left eye demonstrated almost 360° angle closure (Shaffer grading 0-1), while the right eye revealed an open angle. In view of persistent ocular pain despite initial topical IOP-lowering therapy, the patient subsequently underwent therapeutic laser peripheral iridotomy (LPI) at the three and nine o’clock positions (Figure 2A-2B). UBM could not be performed at that time due to the severity of the patient’s symptoms.

**Figure 3:**
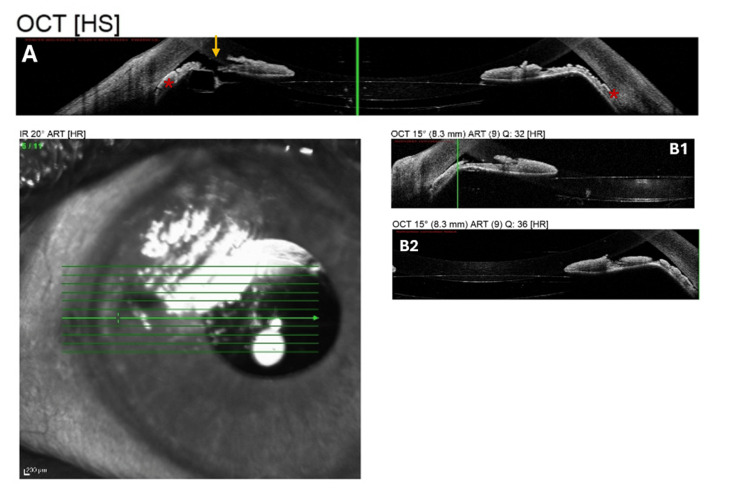
Horizontal AS-OCT scan of the left eye (A) The scan demonstrates bilaterally almost closed anterior chamber angles, with the peripheral iris in close apposition to the inner corneal surface on both nasal and temporal sides (red asterisk). Anterior bowing of the iris is evident, while the IOL is visible posteriorly. The nasal LPI is visible (yellow arrow). (B1) Shallow anterior chamber nasally, anterior bowing of the peripheral iris, and near-total iridotrabecular contact. The IOL is visible posteriorly, and there is no evidence of pupillary block. (B2) Temporal AS-OCT scan demonstrating that the anterior bowing is pronounced and the angle is essentially closed. AS-OCT, anterior segment optical coherence tomography; IOL, intraocular lens; LPI, laser peripheral iridotomy

After LPI, anterior segment optical coherence tomography (AS-OCT) was performed using the Spectralis OCT system (Heidelberg Engineering, Heidelberg, Germany). AS-OCT revealed a shallow anterior chamber with peripheral iris bowing and nearly complete iridotrabecular contact. The two LPIs were visible temporally and nasally, corresponding to the nine and three o’clock positions, ruling out pupillary block as the primary cause of angle closure. Across multiple scan orientations, the iris appeared displaced anteriorly with a posteriorly located IOL, further supporting a posterior pushing mechanism (Figure 3A-3C).

**Figure 4:**
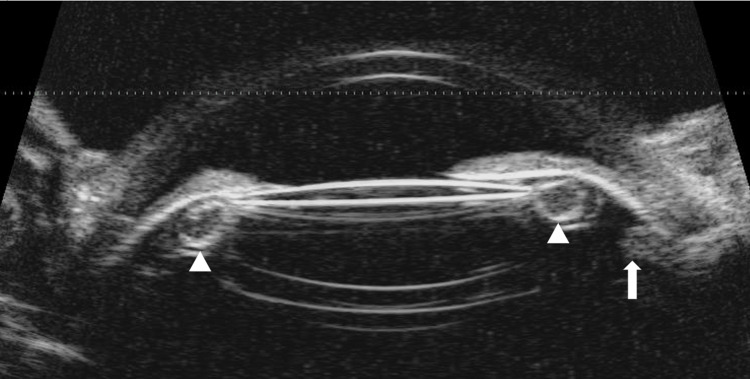
UBM image of the affected eye The ciliary body (white arrow) is rotated anteriorly. A Soemmering’s ring (white arrowheads), which extends from the edge of the IOL optic to the ciliary processes, is displaced anteriorly. IOP, intraocular pressure; UBM, ultrasound biomicroscopy

UBM (Accutome UBM; Keeler) revealed a large hyperechoic structure, seen in direct apposition to the posterior surface of the iris. This 360-degree circumferential contact appeared to push the iris anteriorly, contributing to angle closure and a markedly shallow anterior chamber predominantly in the periphery (Figure 4).

Given the patient’s history of pseudoexfoliative material and the overall anatomical findings, the structure is hypothesized to be a Soemmering’s ring, a retained cortical remnant within the capsular bag. This, combined with zonular weakness from PEX, seems to be pushing the iris forward and narrowing the angle. The anterior displacement of the iris and the persistent angle closure despite the peripheral iridotomies support this pathophysiological mechanism. The absence of uniform shallowing of the anterior chamber and lack of posterior aqueous misdirection on UBM excluded malignant glaucoma.

Despite the initial treatment, at the one-week follow-up, the anterior chamber remained significantly shallow, and IOP rose to 32 mmHg, even under the maximum tolerated topical and systemic IOP-lowering medication. The topical therapy included a beta-blocker (timolol), an alpha-2 adrenergic agonist (brimonidine), a prostaglandin analogue (latanoprost), and a carbonic anhydrase inhibitor (dorzolamide), as well as topical atropine (once daily) and systemic acetazolamide (250 mg twice daily). The persistent elevation of IOP, combined with anterior displacement of the IOL, led to the need for further surgical intervention. The patient was referred to another center, where pars plana vitrectomy was reportedly performed. The patient was subsequently lost to follow-up.

## Discussion

Secondary angle closure in pseudophakic eyes typically arises from mechanisms such as pupillary block, malignant glaucoma, or zonular disruption, but an enlarging Soemmering’s ring represents a distinct and increasingly recognized cause [2,3]. Soemmering’s ring is a common, typically benign complication of cataract surgery defined as a circumferential, doughnut-shaped proliferation of residual lens epithelial cells in the peripheral part of the capsular bag [2,3,8]. Sometimes a Soemmering’s ring can induce angle closure through two primary pathways: traditional pupillary block or a non-pupillary block mechanism involving direct mechanical compression [3,8,11]. In cases without pupillary block, the enlarging Soemmering’s ring acts as a physical mass posterior to the peripheral iris, providing support that wedges the iris against the trabecular meshwork [3,8]. This often results in progressive peripheral anterior synechiae (PAS) and a plateau-like iris configuration that persists even after a patent laser iridotomy, as in our case [1,2,8].

The literature review emphasizes that eyes with PEX are particularly predisposed to this progressive synechial closure. In PEX, zonular fibers are compromised by the production and progressive accumulation of abnormal fibrillar extracellular material [10]. Clinically, these fibers appear encrusted, frayed, distorted, and fragmented [10]. Subclinical anterior chamber inflammation associated with PEX is thought to stimulate the excessive proliferation of residual lens epithelial cells, leading to a massive, pathogenic Soemmering’s ring [1-3]. In addition to this, PEX is characterized by abnormal fibrillar deposits that weaken the zonules [10]. This zonular laxity allows the entire IOL-capsule-Soemmering’s ring complex to shift anteriorly, facilitating iridocorneal contact and mechanical angle crowding, as in our case [10]. Reports have specifically described patients with PEX who developed 360° PAS progression over several years as the Soemmering’s ring enlarged, despite having patent iridotomies and moderately deep central anterior chambers [2].

For patients presenting with progressive angle closure after cataract surgery, high-frequency UBM is an indispensable diagnostic tool because it provides high-resolution, in vivo visualization of anterior segment structures posterior to the iris that remain hidden during standard slit-lamp biomicroscopy [6,7]. UBM allows clinicians to qualitatively and quantitatively evaluate the anatomic relationships necessary to differentiate between complex mechanisms such as pupillary block, malignant glaucoma, and plateau iris configuration [5-7].

Soemmering’s ring often appears as a "string of sausages" or a "donut shape" [3,5]. UBM reveals the proliferation as a large, hyperechoic, ovoid or ellipsoid mass behind the peripheral iris, which is essential for diagnosis since these rings often remain undetected clinically if the central posterior capsule is clear [1-3,5,11]. The technology specifically clarifies how an enlarged Soemmering’s ring can provide posterior support that physically wedges the iris root against the trabecular meshwork, facilitating progressive synechial closure even after a patent iridotomy has been performed [1-3,8]. For patients with PEX, UBM is particularly valuable for detecting zonular instability and the anterior rotation of ciliary processes that frequently coexist with pathogenic ring growth [1]. UBM is essential also to determine if the closure is due to pupillary block (indicated by iris bombé), a non-pupillary block mechanism (where the Soemmering’s ring provides direct posterior support to the iris), or malignant glaucoma (uniform shallowing of the anterior chamber, where usually the central portion of the anterior lens capsule is nearly in contact with the cornea) [3,5]. In eyes with PEX, imaging also helps identify zonular instability or the anterior rotation of ciliary processes that may exacerbate the condition [1,10].

AS-OCT can also play a supportive role since it provides high-resolution images of the iridocorneal angle and can clearly show a planar iris configuration versus iris bombé, helping to rule out or confirm pupillary block [2,8].

Initial management of secondary angle closure in the presence of a Soemmering’s ring focuses on rapid IOP reduction with aqueous suppressants, including topical beta-blockers, carbonic anhydrase inhibitors, and alpha-agonists [4,9]. When a pupillary block component is suspected, LPI represents the first-line intervention [2,6,10]. If LPI alone fails to adequately deepen the anterior chamber or relieve IOP elevation, Nd:YAG laser capsulotomy performed through the existing iridotomy can disrupt the loculated Soemmering’s ring or posterior capsule, allowing posterior displacement of the IOL-capsular complex and restoration of aqueous flow [1,5].

The timeline for symptoms to manifest ranges from a few years to several decades [2,3,5,8]. Surgical management is indicated when LPI fails to control IOP or deepen the chamber, with surgical removal of the Soemmering’s ring, which resolves the physical obstruction, or with IOL exchange and vitrectomy, especially in cases complicated by uveitis-glaucoma-hyphema (UGH) [3,9].

## Conclusions

While pupillary block is the most common cause of angle closure after cataract surgery, an enlarging Soemmering’s ring introduces a distinct non-pupillary block mechanism. In eyes with PEX, subclinical inflammation may stimulate excessive growth of the Soemmering’s ring. Furthermore, zonular instability, a characteristic of PEX, allows the entire IOL-capsule-Soemmering’s ring complex to shift anteriorly, worsening the crowding of the angle. UBM is essential in the evaluation of suspected Soemmering’s ring-related angle closure, as it allows direct visualization of posterior chamber structures that cannot be assessed with routine slit-lamp examination. It confirms the presence of the ring, helps clarify the underlying mechanism of angle closure, and provides valuable guidance for management.
